# Association between current major depressive episode and the occurrence of menstrual irregularities in the last six months in university students in Lima, Peru

**DOI:** 10.17843/rpmesp.2025.424.14992

**Published:** 2025-12-11

**Authors:** Ximena Pantoja-Coronel, Gianella Castillón-Véliz, Valeria Ninanya-Cruz, Gema Roca Terry, Yvana Morales-Sánchez, Fabián Fiestas

**Affiliations:** 1 Universidad de Ciencias Aplicadas, Lima, Perú. Universidad Peruana de Ciencias Aplicadas Universidad de Ciencias Aplicadas Lima Peru

**Keywords:** Menstruation Disturbances, Amenorrhea, Oligomenorrhea, Depressive Disorder Major

## Abstract

**Objectives.:**

To evaluate whether there is an association between current major depressive episode (CMDE) and menstrual irregularities (MI), including amenorrhea, oligomenorrhea, or polymenorrhea, in Peruvian university students in 2024.

**Materials and methods.:**

An observational, cross-sectional, and analytical study was conducted on students from a private university in Lima, Peru. The Patient Health Questionnaire 9 (PHQ-9) was used to identify a CMDE, and a survey with closed-ended questions was used to identify cases of MI. The measure of association was the prevalence ratio (PR), derived from Poisson regression models, both crude and adjusted for potential confounding factors.

**Results.:**

A total of 250 women between 18 and 30 years of age participated (median age: 20 years, interquartile range from 19 to 21 years). 73.2% presented at least one MI (amenorrhea, oligomenorrhea, or polymenorrhea) and 70.8% had CMDE of any grade (29.2% mild and 41.6% moderate-severe). Compared to students without CMDE, the occurrence of any MI was more likely in those students with moderate-severe CMDE (aPR=1.33; 95% CI: 1.08-1.62; p=0.006), but was not different regarding those with mild CMDE (aPR=1.20; 95% CI: 0.95-1.5; p=0.112).

**Conclusions.:**

MIs and CMDE are frequent in university students. Moderate-severe CMDE was associated with a higher risk of MI. Future longitudinal studies will allow for the establishment of temporality and other relevant aspects to assess a possible causal relationship between MI and CMDE, including the specific relationship with each type of MI.

## INTRODUCTION

Menstrual irregularity (MI) is considerably common. Globally, the prevalence of irregular menstrual cycles varies between 5% and 35.6% and varies according to age, country of origin, and occupation ^1^. The most frequent menstrual irregularities are amenorrhea, oligomenorrhea, and polymenorrhea ^2^. In Peru, no epidemiological studies were found regarding the magnitude of this problem or its localization in the population experience, in terms of space, time, and person.

For their part, mood disorders and major depressive disorder are also prevalent health problems in Peru, especially among adolescents and young adults. In 2023, the Ministry of Health (MINSA) attended 280,917 cases of depression, the majority of which were women (75.5%) and 16.5% were under 18 years of age ^3^. Likewise, an epidemiological study in the general population, carried out in 2005 in five Peruvian cities of the coast, highlands, and jungle, found that major depressive disorder had an annual prevalence of 2.7% among adults, placing depression among the most common mental disorders in the country ^4^. This high prevalence of depression explains the fact that this disorder is among the top 10 causes of disease burden in Peru. Specifically, for 2019, the Peruvian Ministry of Health reported that unipolar depression caused between 150,000 and 200,000 years of life lost due to disability or premature death ^5^.

The extent and relationship of depression with multiple physical health problems of the person make this a condition highly suspected of contributing to the causal pathway of diverse chronic physical disorders, especially those with a high underlying hormonal pathophysiological load, such as MIs. The relationship between major depressive disorder and MI has some empirical evidence. On the one hand, it has been found that during depression the hypothalamic-pituitary-adrenal (HPA) axis is activated, which leads to the inhibition of reproductive hormones and, as a consequence, to an alteration in ovarian function ^6^. Added to these findings at the hormonal level are the results of a study conducted in South Korea, where it was found that women with irregular cycles had greater depressive symptoms ^7^.

Major depressive disorder and menstrual irregularities have been studied extensively separately, but the association between them has been very sparsely investigated. In fact, establishing a causal connection between both entities would have important implications for both the prevention and management of these health problems so common among women of reproductive age. Thus, the present study had the objective of evaluating whether there is an association between the occurrence of current major depressive episode and MIs in young adult, Peruvian female university students.

**KEY MESSAGES**

Motivation for conducting the study. Depression and menstrual irregularities (MI) are common, and a causal relationship between both conditions is plausible given their neuro-hormonal nature.

Main findings. The present study found that, compared to women without a current major depressive episode (CMDE), those with a moderate-to-severe CMDE have a higher probability of presenting with some form of MI. On the other hand, women with mild CMDE did not differ from those without CMDE regarding the probability of MI.

Public health implications. The results support the causal hypothesis, but longitudinal studies are required to confirm if depression increases the risk of MI. If corroborated, young women with CMDE should receive preventive gynecological care oriented toward the management of MI.

## MATERIALS AND METHODS

### Design

An observational, cross-sectional, and analytical study was conducted.

### Population

The study population included female students from a private university in Lima, Peru, in the period from May to June 2024. The inclusion criteria were being between 18-30 years old. Pregnant or lactating women, or those with primary amenorrhea, and those using drugs such as hormonal contraceptives, non-steroidal anti-inflammatory drugs (NSAIDs), and anticoagulants chronically and constantly for a minimum time of 6 weeks were excluded.

### Definition of variables

The exposure variable is the occurrence of current major depressive episode (CMDE), measured with the Patient Health Questionnaire (PHQ-9) scale. The outcome variable is the occurrence of any menstrual irregularity, including amenorrhea, oligomenorrhea, or polymenorrhea. Age, university major, and history of diagnosis of any mental health disorder (bipolar disorder, borderline personality disorder, and generalized anxiety disorder) were included as variables with a potential confounding effect. Other confounding variables considered were the presence of any endocrine disorder (polycystic ovary syndrome, hyperprolactinemia, premature menopause, and hypothyroidism), any eating disorder (anorexia nervosa, bulimia nervosa, and orthorexia); and whether the woman practices any competitive sport (supplementary material).

For the independent, or exposure, variable, a sum of the score obtained for each item of the PHQ-9 was performed. Based on this, two types of variables were generated, one dichotomous and the other with three categories. The dichotomous variable identified those who did not present the CMDE (coded as zero) and those who did (coded as 1). For its part, the variable where the CMDE is classified into three categories included the category without CMDE (coded as zero), the category of mild CMDE (coded as 1), and that of moderate-severe CMDE (coded as 2). For the dependent or outcome variable, if a participant answered “Yes” to any of the questions coding for amenorrhea, oligomenorrhea, and polymenorrhea, it was considered as MI present, coded as 1, and 0 if she did not report any of the three conditions.

For a better understanding of the definition and categorization of each variable, the supplementary material can be consulted.

### Sample

The sample calculation was performed with the EPIDAT 4.2 program. The dependent variable was taken as a dichotomous categorical variable of nominal scale, so an independent comparison of proportions was performed. To perform the calculation, we took data from the study by Maurya *et al.* (2022), which found that of 1,334 women who had menstrual irregularities, 376 (28%) had depression. Furthermore, it was found that of 11,373 women who did not have menstrual irregularities, 1,638 (14%) had depression ^8^. For the appropriate calculation, we used a confidence level of 95% and a power of 80%. Although the sample size calculation resulted in 264 people with these assumptions, with 132 for each group, 250 people with valid surveys were finally recruited. The type of sampling was non-probabilistic by convenience.

### Instruments

The questionnaire presented 19 questions that were divided into two sections: Section 1: general data (age and faculty), menstrual irregularity (amenorrhea, oligomenorrhea, or polymenorrhea), and confounding factors (history of diagnosis of any mental health disorder, of any endocrine disorder, of any eating disorder, and the practice of any competitive sport currently); and Section 2: current major depressive episode.

For the MI section, items from the questionnaire developed by Shapley *et al.* (2004) were used to identify the occurrence of oligomenorrhea, polymenorrhea, and amenorrhea. The questions used were the following: “In the last 6 months, have you had menstrual periods with less than 21 days (3 weeks) between one period and another?”, for polymenorrhea; “In the last 6 months, have you had menstrual periods with more than 35 days (5 weeks) between one period and another?”, for oligomenorrhea; and “Have you had a menstrual period in the last 6 months?”, for amenorrhea ^9^. This section of the questionnaire was submitted, for the purposes of this research, to expert judgment with two gynecologists and an endocrinologist to validate the accuracy of the translation into Spanish. They were presented with both the version in its original language (English) and the translation performed by the research group, so that they could make the due comparison. After an exhaustive review, in which they verified that the meaning of the question was not altered when translated into Spanish, they unanimously validated the translation of the instrument.

For the second section, the PHQ-9 scale was used, a self-report questionnaire based on 9 criteria for major depressive disorder according to the DSM-4, which remain valid in the DSM-5. This instrument evaluates depressive symptoms during the last two weeks, rating them on a 4-point Likert-type scale ^10^^,^^11^. As shown in Annex 2, for the purposes of the present study, a score of 0 to 9 has been considered as “CMDE absent”, 10 to 14 as “mild CMDE”, and 15 to 27 as “moderate-severe CMDE”; considering that the cut-off point to diagnose CMDE is greater than or equal to 10, whose sensitivity and specificity is 88% (10). The reliability of this questionnaire was validated in Peru by Villarreal-Zegarra et al. in 2023, in which good psychometric properties were determined regarding structural validity (comparative fit index [CFI]=0.974) and internal consistency (α=0.89; ω=0.86) ^12^.

### Data collection

Participants were selected through non-probabilistic sampling on the campus of a private university in Lima, Peru, during the period from May 27 to June 6, 2024. The fieldwork was carried out by five researchers (XPC, GCV, VNC, GRT, and YM), who organized themselves into groups to enter classrooms, hallways, and study rooms, where they invited students to participate in the study. Those who showed interest were provided with a QR code that directed to the informed consent. After reading it, participants could access the questionnaire only if they pressed the “accept” button, thus indicating their consent. The research team remained present during the completion of the online questionnaire, although the participant responded from her own mobile phone, confidentially. In this way, the correct understanding of the questionnaire and a uniform application among participants were guaranteed. The online completion of the questionnaire was carried out in Google Forms and lasted approximately 8 minutes.

### Analysis plan

The analysis of the collected data was performed using the statistical package STATA ® version 17. Categorical variables (major depressive disorder, secondary amenorrhea, oligomenorrhea, and polymenorrhea) were analyzed using absolute frequencies and proportions. On the other hand, the numerical variable (age) was analyzed using measures of central tendency (median) and the corresponding measure of dispersion (interquartile range). Bivariate analyses were performed using Chi-square and Mann-Whitney U test. Likewise, association analyses between the independent and dependent variables were performed using crude and adjusted Poisson regression, taking into account the potential effect of confounding variables. The selection of variables included in the adjusted model was carried out based on a theoretical criterion, supported by scientific literature and the biological plausibility of their relationship with the main study variables. Those variables that, according to previous evidence, could act as confounding factors between MI and CMDE were included. Two types of Poisson regression modeling were performed: In modeling type 1, both crude and adjusted, the depression variable was considered as dichotomous: with and without CMDE (as planned when the sample size calculation was made); and in modeling type 2, crude and adjusted, where the depression variable was considered with three categories: CMDE absent, mild CMDE, and moderate-severe CMDE. A value of p<0.05 was considered significant. A statistical exploration was performed to identify collinearity between the variables considered in the adjusted model through the variance inflation factor (VIF).

### Ethics and informed consent

The research project was approved by the ethics committee of the Universidad Peruana de Ciencias Aplicadas (PI 134-24). The principles of non-maleficence, beneficence, justice, and autonomy were followed. Participants were informed verbally and in writing that they had total freedom to withdraw during the data collection process, without any type of consequence. Likewise, the surveys were conducted anonymously.

## RESULTS

### Sample selection

**Figure 1:**
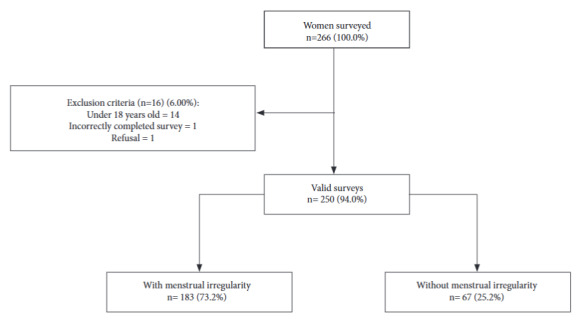
Flowchart of participant selection.

Two hundred sixty-six female students were invited to participate, of whom one rejected the invitation. Of the rest, 14 were excluded for being under 18 years of age and one for having completed the survey incorrectly. Thus, the sample consisted of 250 women (Figure 1).

### Epidemiological and clinical characteristics of the sample

The characteristics of the sample are presented in Table 1. The median age of the participants was 20 years (IQR: 19-21), with 99% of them being between 18 and 26 years old. Regarding the faculty of studies, the majority (36%) belonged to liberal arts and humanities majors (Communication, Law, Education, Human Sciences, Design, and Contemporary Arts) followed by 30.8% who were students of health careers (Human Medicine, Nutrition and Dietetics, and Physical Therapy). Regarding history, 67 participants (26.8%) reported having had a diagnosis of a mental health disorder at some point in their life (bipolar disorder, borderline personality disorder, and generalized anxiety disorder) and 69 (27.6%) having had a diagnosis of an endocrine disorder (polycystic ovary syndrome, hyperprolactinemia, premature menopause, and hypothyroidism). In addition, 32 participants (12.8%) have had a diagnosis of an eating disorder and 42 participants (16.8%) practiced a competitive sport at the time of the survey. The majority of participants presented at least one MI in the last six months, totaling 183 (73.2%). Within menstrual irregularities, the most frequent was polymenorrhea, which occurred in 115 participants (46%); in second place, oligomenorrhea occurred in 108 participants (43.2%); and in third place, amenorrhea in 9 participants (3.6%). Regarding the presence of current major depressive episode, evaluated according to the PHQ-9, a total of 73 participants (29.2%) did not show scores indicating the presence of said disorder, 73 participants (29.9%) presented scores corresponding to mild depression, while 104 participants (41.6%) had a moderate-severe score.

**Table 1:** Characteristics of the participants.

Characteristics		n=250	%
Age (years) ^a^		20	19.2
Faculty			
	Health	77	30.8
	Letters and Humanities	90	36.0
	Engineering and Architecture	49	19.6
	Economics, Business, and Administration	34	13.6
History of mental health disorder diagnosis ever in life ^b^		67	26.8
History of endocrine disorder diagnosis ever in life ^c^		69	27.6
History of eating disorder diagnosis ever in life ^d^		32	12.8
Currently practicing competitive sport		42	16.8
Any menstrual irregularity (amenorrhea, oligomenorrhea, or polymenorrhea)			
	Yes	183	73.2
	No	67	26.8
Menstrual irregularities ^e^			
	Amenorrhea	9	3.6
	OOligomenorrhea	108	43.2
	Polymenorrhea	115	46.0
	Polymenorrhea and oligomenorrhea	48	19.2
	Polymenorrhea and amenorrhea	1	0.4
Current major depressive episode			
	Absent (PHQ-9 <10)	73	29.2
	Yes, Mild (PHQ-9 10-14)	73	29.2
	Yes, Moderate-severe (PHQ-9 15-25)	104	41.6

a Median and interquartile range. The minimum age was 18 and the maximum was 29 years.

b Bipolar disorder, borderline personality disorder, and generalized anxiety disorder.

c Polycystic ovary syndrome, hyperprolactinemia, premature menopause, and hypothyroidism.

d Anorexia nervosa, bulimia nervosa, and orthorexia.

e Cases with more than one MI in the last six months may be present, so percentages will not sum to 100%.

### Association between MI and CMDE

Table 2 shows a statistically significant association between CMDE and any MI (p=0.012). Specifically, of the participants who had mild CMDE, 72.6% had at least one of the types of MI, while this proportion was 81.7% in women who presented moderate-severe CMDE. This percentage was 61.6% among women without CMDE. On the other hand, no association was found between MI and the rest of the independent variables such as age (p=0.582), university major (p=0.160), history of mental health disorder (p=0.110), history of diagnosis of endocrine disorder (p=0.151), history of diagnosis of eating disorder (p=0.501), and the practice of competitive sports (p=0.631).

**Table 2:** Association between menstrual irregularities in the last six months and current major depressive disorder and covariates.

Variables		Menstrual Irregularity		p-value^b^
		No n=67 (%)	Yes n=183 (%)	
Current Major Depressive Episode				
	Absent	28 (38.4)	45 (61.6)	0.012
	Yes, Mild	20 (27.4)	53 (72.6)	
	Yes, Moderate-severe	19 (18.3)	85 (81.7)	
Age (years)		20 (18-21)*	20 (19-21)^a^	0.582
Faculty				
	Health	27 (35.1)	50 (64.9)	0.160
	Letters and Humanities	20 (22.2)	70 (77.8)	
	Engineering and Architecture	14 (28.6)	35 (71.4)	
	Economics, Business, and Administration	6 (17.7)	28 (83.4)	
History of mental health disorder diagnosis ever in life				
	No	54 (29.5)	129 (70.5)	0.110
	Yes	13 (19.4)	54 (80.6)	
History of endocrine disorder diagnosis ever in life				
	No	53 (29.3)	128 (70.7)	0.151
	Yes	14 (20.3)	55 (79.7)	
History of eating disorder diagnosis ever in life				
	No	60 (27.5)	158 (72.5)	0.501
	Yes	7 (21.9)	25 (78.1)	
Currently practicing competitive sport				
	No	57 (27.4)	151 (72.6)	0.631
	Yes	10 (26.8)	32 (73.2)	

a Median and interquartile range. The minimum age was 18 and the maximum was 29 years.

b For categorical variables, the Chi-squared test was used, and for the numerical variable age, the Wilcoxon rank-sum test was used.

In Table 3, the results of the Poisson regression analyses are shown. In modeling type 1, it was found that CMDE was associated with having MI, both in the crude model (cPR=1.26; 95% CI: 1.04‒1.54; p=0.020) and in the adjusted one (aPR=1.25; 95% CI: 1.02‒1.53; p=0.034). In modeling type 2, no statistically significant association was found between mild CMDE and menstrual irregularities in the simple regression nor in the adjusted model. However, it was observed that the prevalence of menstrual irregularities in women with symptoms of moderate-severe CMDE was 1.28 times the prevalence of the same among women without said disorder (PR=1.33; 95% CI: 1.08‒1.62; p-value=0.006). This association remained statistically significant even after adjustment for age, sex, history of mental disorders, endocrine disorders, eating disorders, and current practice of competitive sport (aPR=1.28; 95% CI: 1.03‒1.58; p=0.022).

**Table 3:** Association between current major depressive episode and menstrual irregularities: Crude and adjusted models.

Variable		Crude Association			Adjusted Association		
		cPR	95% CI	***p*-value**	aPR^a^	95% CI	***p*-value**
Current Major Depressive Episode (two categories)							
	No depression	1			1		
	With depression	1.26	1.04‒1.54	0.020	1.25	1.02‒1.53	0.034
Current Major Depressive Episode (three categories)							
	Absent	1			1		
	Mild	1.78	0.94‒1.48	0.163	1.20	0.95‒1.5	0.112
	Moderate-severe	1.33	1.08‒1.62	0.006	1.28	1.03‒1.58	0.022

cPR: crude prevalence ratio, aPR: adjusted prevalence ratio, CI: confidence interval.

a Adjusted for age, faculty, history of mental health disorder diagnosis (bipolar disorder, borderline personality disorder, and generalized anxiety disorder), of an endocrine disorder (polycystic ovary syndrome, hyperprolactinemia, premature menopause, and hypothyroidism), and of an eating disorder (anorexia nervosa, bulimia nervosa, and orthorexia), and currently practicing competitive sport.

Exploratorily, the relationship between the presence of CMDE and each of the three menstrual irregularities studied, taken independently of one another, was also assessed (Table 4). The association between amenorrhea and the current major depressive episode was not estimable because the sample size was limited, given that of the nine women with amenorrhea, all resulted with CMDE. For its part, oligomenorrhea was not found to be associated with CMDE, both in the crude and adjusted analysis. Additionally, it was found that the proportion of polymenorrhea in women with mild CMDE was 1.5 times the proportion of the same among women without said disorder. This association remained statistically significant even after adjustment for potential confounding factors (aPR=1.6; 95% CI: 1.0‒2.3; p=0.032).

**Table 4:** Association between current major depressive episode and oligomenorrhea and polymenorrhea: Crude and adjusted model

	Variable	Crude Association			Adjusted Association		
		cPR	95% CI	***p*-value**	aPR^a^	95% CI	***p*-value**
Oligomenorrhea	Current major depressive episode						
Absent	1	-	-	1	-	-
Mild	1	0**.**7‒1**.**5	1	1	0**.**7‒1**.**5	0**.**874
Moderate-severe	1**.**1	0**.**8‒1**.**6	0**.**510	1**.**1	0**.**7‒1**.**5	0**.**761
Polymenorrhea	Current major depressive episode						
	Absent	1	-	-	1	-	-
	Mild	1**.**5	1**.**0‒2**.**3	0**.**034	1**.**6	1**.**0‒2**.**3	0**.**032
	Moderate-severe	16	1**.**1‒2**.**3	0**.**018	1**.**5	0**.**9‒2**.**2	0**.**066

cPR: crude prevalence ratio, aPR: adjusted prevalence ratio, CI: confidence interval.

a Adjusted for age, faculty, history of mental health disorder diagnosis (bipolar disorder, borderline personality disorder, and generalized anxiety disorder), of an endocrine disorder (polycystic ovary syndrome, hyperprolactinemia, premature menopause, and hypothyroidism), and of an eating disorder (anorexia nervosa, bulimia nervosa, and orthorexia), and currently practicing competitive sport.

Finally, an association was found between women with moderate-severe CMDE and the presence of polymenorrhea in the simple regression (PR=1.6; 95% CI: 1.1‒2.3; p=0.018). However, this association did not remain statistically significant at an alpha equal to 0.05, probably due to an insufficient sample size to sustain the multivariate model (aPR=1.5; 95% CI: 0.9‒2.2; p=0.066). For all models, the VIF resulted in less than 2 for all variables considered in the multivariate models.

## DISCUSSION

The main finding of this study is that, compared to female university students without CMDE, the occurrence of at least one MI (amenorrhea, polymenorrhea, or oligomenorrhea) in the last six months was more frequent in those with CMDE, independently of potential confounding variables. Likewise, it was found that the association between MI and CMDE occurs specifically for those with moderate-severe CMDE and not for those with mild CMDE, given that the latter did not differ in the probability of having MI from those who did not present CMDE.

Additionally, this study has found that the most frequent MI in the studied population was polymenorrhea. Furthermore, a significant proportion (70.8%) of participants presented CMDE, with a majority showing moderate-severe symptoms. No association was found between oligomenorrhea or amenorrhea and CMDE of any grade, but it was found between polymenorrhea and mild CMDE.

Although the quantity of studies on this topic is scarce, especially in Latin America, our results are consistent with previous investigations. For example, Kim *et al.* (2017) found that the number of menstrual problems correlates with depression ^13^. Said study focused on female defectors from North Korea and refugees in South Korea, who faced multiple stressors such as unfavorable health conditions, torture, detention, and arrest during their escape, in addition to problems of cultural adaptation and radical changes in their lifestyle, which may have led to elevated levels of stress and anxiety and subsequently to menstrual problems (such as amenorrhea, hypomenorrhea, menorrhagia, polymenorrhea, oligomenorrhea, changes in the amount of menstrual flow, and changes in the amount of blood clots). Among the limitations of said study is the small sample size (n=126). Similarly, Jung and Pang (2023), in a study developed in the population of South Korea, found that the sum of the depressive symptom score was higher in women with some menstrual irregularity compared to those without depression ^7^. Likewise, Maurya *et al.* (2020) found an association between MIs and depression in its different levels of severity (mild, moderate, and severe) ^8^ in a sample of 12,707 adolescents from the states of Uttar Pradesh and Bihar in India. Thus, our study, carried out in young adult women from a Latin American country, with an urban lifestyle and homogeneous regarding socioeconomic, educational, and cultural level, finds results that support the causal hypothesis between depression and menstrual alterations. Furthermore, our findings shed light on the way in which this relationship may occur by finding that, when compared to not having CMDE, it is moderate-severe CMDE, and not mild, that is associated with having some MI. In this sense, future studies could assess the role of environmental aspects, such as academic load, in the relationship between CMDE and MI in university students.

However, despite this evidence relating depression to menstrual cycle problems, it is still premature to propose that a causal correlation actually exists. In fact, there are studies with results inconsistent with what was found in our study. For example, Bisaga *et al*. (2002), in a study carried out in the United States, did not evidence an association between polymenorrhea and the presence of depressive symptoms ^14^. Furthermore, to interpret that a statistical association between two variables is causal, the temporality with which the variables are related must necessarily be established. This is yet to be explored in longitudinal studies, given that our study had a cross-sectional design.

A causal relationship between depression and menstrual cycle alterations is biologically plausible. The menstrual cycle is regulated by the endocrine system, and this can be affected in depressive states. In depression, persistent stress activates the hypothalamic-pituitary-adrenal axis, which causes a greater release of CRH, ACTH, and cortisol. This hormonal imbalance can alter the regulation of the menstrual cycle, affecting its regularity and function ^6^. Thus, the result of our study is consistent with the causal hypothesis, although, given its cross-sectional design, it was not possible to establish that the CMDE was prior to the MI, as would be necessary to demonstrate to sustain that the former causes the latter.

Regarding the frequency of MI, our results are consistent with the studies carried out by Kim et al., where the menstrual irregularity with the highest prevalence was polymenorrhea ^13^. This can be explained because the shortened cycle is more easily recognized by the female population. Another similarity was the high frequency of CMDE, which agrees with the study by Maldonado et al., with a frequency of 65.5% in a similar population of university students ^15^. This may be due to biological and psychosocial factors, which can predispose to affective disorders in urban and university populations ^16^. On the other hand, it is noteworthy that our results referring to the frequency of MI differ from what was found in the study by Mittiku et al. (2022). These authors found a prevalence of menstrual irregularities (33.4%) in students from Ethiopia ^17^, which means a prevalence quite lower than what was found in our study (73.2%). This can be attributed to socioeconomic and cultural differences between both countries, and to methodological differences in relation to the criteria to define MI.

The present study is not exempt from limitations. Among the main ones is the impossibility of establishing temporality between variables due to the cross-sectional design that prevents determining if depression is prior to MI. Another limitation refers to the fact that the sample size reached was 250 people, which is 5% less than the 264 stipulated by the original sample calculation. Since this gap is small, it did not have a large impact on the statistical power to address the primary objective of this study. However, the sample size was indeed limited regarding the exploratory statistical analyses to assess the association between the levels of severity of CMDE and each of the three MIs studied. For example, only 9 women reported amenorrhea, and all of them had CMDE, which prevented verifying the statistical association between both variables. Likewise, the cross-sectional design with non-probabilistic sampling imposes a risk of selection bias that limits the validity of the results, especially regarding the estimation of the prevalence of both MI and CMDE as it is not a representative sample of the population of female university students in Peru. Future studies with longitudinal design and larger sample size are necessary to confirm the temporality between MI and CMDE, control for selection biases, and also establish if the relationship is specific for one or more of the types of MI. On the other hand, the use of self-report surveys could introduce information and recall biases, which could be reduced if data are collected in real-time by clinical specialists systematically in future longitudinal studies. Notwithstanding these limitations, our study explores a sparsely studied topic, especially in the Latin American female population, and given its findings, makes it reasonable to continue deepening the study of the potential relationship between depression and MIs, especially with longitudinal methodologies.

In conclusion, young adult women with a current major depressive episode of moderate-severe grade have a higher probability of presenting with some menstrual irregularity in the last six months compared to those without said depressive episode. This was not the case regarding women with mild depression, who had a similar probability of having some MI as women without a depressive episode. Future investigations with a larger sample size and longitudinal design will allow for clarifying aspects still pending regarding the possible causal relationship between CMDE and the distinct MIs.
