# Supplementary material for: Association between current major depressive episode and the occurrence of menstrual irregularities in the last six months in university students in Lima, Peru
Source: Rev Peru Med Exp Salud Publica. 2025 Dec 11;42(4):400–8. doi: 10.17843/rpmesp.2025.424.14992 (PMC12879977; doi:10.17843/rpmesp.2025.424.14992)
Supplement: Supplementary material. — Available in the electronic version of the RPMESP. [file rpmesp-42-04-14992-s001.docx]

# **MATERIAL SUPLEMENTARIO**

1. **Anexo 1**

| **INSTRUMENTOS** | |
| --- | --- |
| **Sección 1.1: Variables sociodemográficas y de salud** | |
| Edad | ¿Cuál es tu edad? |
| Facultad | ¿A qué facultad perteneces?   - Administración en Hotelería y Turismo - Arquitectura - Artes Contemporáneas - Ciencias de la Salud - Ciencias Humanas - Comunicaciones - Derecho - Diseño - Economía - Educación - Ingeniería - Negocios - Psicología |
| Trastornos de salud mental | ¿Algún médico le ha diagnosticado alguno de los siguientes trastornos de salud mental: Trastorno bipolar, Trastorno de personalidad y Trastorno de ansiedad generalizada?   - Si - No |
| Trastornos endocrinos | ¿Algún médico le ha diagnosticado alguno de los siguientes trastornos endocrinos: Síndrome de ovario poliquístico, Hiperprolactinemia, Menopausia precoz e Hipotiroidismo?   - Sí - No |
| Trastornos de conducta alimentaria | ¿Ha sido diagnosticada con algún trastorno de la conducta alimentaria (Anorexia Nerviosa, Bulimia Nerviosa, Trastorno por atracón, Ortorexia)?   - Sí - No |
| Deportes de competición | ¿Actualmente, practicas algún deporte competitivo de manera profesional (correr maratones, jugar baloncesto, voley o fútbol, o participar en competencias de boxeo, judo o taekwondo)?   - Sí - No |
| **Sección 1.2: Irregularidad menstrual** | |
| Amenorrea secundaria | ¿Ha tenido un período menstrual en los últimos 6 meses?   - Sí - No |
| Polimenorrea | En los últimos 6 meses, ¿ha tenido períodos menstruales con menos de 21 días (3 semanas) entre un período y otro?   - Sí - No |
| Oligomenorrea | En los últimos 6 meses, ¿ha tenido períodos menstruales con más de 35 días (5 semanas) entre un periodo y otro?   - Sí - No |
| **Sección 2 PHQ9** | |
| Durante las últimas 2 semanas, ¿con qué frecuencia le han molestado los siguientes problemas? | |
| Tener poco interés o placer en hacer las cosas  0. Nunca  1. Varios días  2. Más de la mitad de los días  3. Casi todos los días | |
| Sentirse desanimada, deprimida o sin esperanza  0. Nunca  1. Varios días  2. Más de la mitad de los días  3. Casi todos los días | |
| Problemas para dormirse, mantenerse dormida o dormir demasiado  0. Nunca  1. Varios días  2. Más de la mitad de los días  3. Casi todos los días | |
| Sentirse cansada o tener poca energía  0. Nunca  1. Varios días  2. Más de la mitad de los días  3. Casi todos los días | |
| Tener poco apetito o comer en exceso*  0. Nunca  1. Varios días  2. Más de la mitad de los días  3. Casi todos los días | |
| Sentir falta de amor propio, que sea un fracaso o que decepciona a sí misma o a su familia  0. Nunca  1. Varios días  2. Más de la mitad de los días  3. Casi todos los días | |
| Tener problemas para concentrarse en cosas tales como leer un libro o ver la televisión  0. Nunca  1. Varios días  2. Más de la mitad de los días  3. Casi todos los días | |
| Se mueve o habla tan lento que otras personas podrían darse cuenta, o al contrario, está tan agitada o inquieta que se mueve mucho más de lo acostumbrado  0. Nunca  1. Varios días  2. Más de la mitad de los días  3. Casi todos los días | |
| Ha tenido pensamientos de que sería mejor estar muerta o hacerse daño de alguna manera  0. Nunca  1. Varios días  2. Más de la mitad de los días  3. Casi todos los días | |
| Si usted se identificó con cualquier problema en este cuestionario, ¿qué tan difícil se le ha hecho complir con su trabajo, atender su casa o relacionarse con otras personas debido a estos problemas?   - Nada en absoluto - Algo difícil - Muy difícil - Extremadamente difícil | |

1. **Anexo 2**

| **Variable** | **Definición conceptual** | **Definición operativa** | **Tipo según su relación** | **Tipo**  **según su naturaleza** | **Escala de medición** | **Valores** | **Fuentes de medición** |
| --- | --- | --- | --- | --- | --- | --- | --- |
| **Episodio depresivo mayor actual (2 categorías)** | A. Cinco (o más) de los síntomas siguientes han estado presentes durante el mismo período de dos semanas y representan un cambio de funcionamiento previo; al menos uno de los síntomas es (1) estado de ánimo depresivo o (2) pérdida de interés o de placer  1. Estado de ánimo deprimido la mayor parte del día, casi todos los días, según se desprende de la información subjetiva o de la observación por parte de otras personas.  (Nota: en niños y adolescentes, el estado de ánimo puede ser irritable).  2. Disminución importante del interés o el placer por todas o casi todas las actividades  la mayor parte del día, casi todos los días (como se desprende de la información  subjetiva o de la observación).  3. Pérdida importante de peso sin hacer dieta o aumento de peso, o disminución o  aumento del apetito casi todos los días. (Nota: en los niños, considerar el fracaso  para el aumento de peso esperado).  4. Insomnio o hipersomnia casi todos los días.  5. Agitación o enlentecimiento psicomotor casi todos los días.  6. Fatiga o pérdida de energía casi todos los días.  7. Sentimiento de inutilidad o culpabilidad excesiva o inapropiada (que puede ser delirante) casi todos los días (no simplemente el autorreproche o culpa por estar enfermo).  8. Disminución de la capacidad para pensar o concentrarse, o para tomar decisiones,  casi todos los días (a partir de la información subjetiva o de la observación por parte  de otras personas).  9. Pensamientos de muerte recurrentes (no solo miedo a morir), ideas suicidas recurrentes sin un plan determinado, intento de suicidio o un plan específico para llevarlo  a cabo.  B. Los síntomas causan malestar clínicamente significativo o deterioro en lo social, laboral u  otras áreas importantes del funcionamiento.  C. El episodio no se puede atribuir a los efectos fisiológicos de una sustancia o de otra afección médica.  D. El episodio de depresión mayor no se explica mejor por un trastorno esquizoafectivo,  esquizofrenia, trastorno esquizofreniforme, trastorno delirante, u otro trastorno especificado o no especificado del espectro de la esquizofrenia y otros trastornos psicóticos.  E. Nunca ha habido un episodio maníaco o hipomaníaco. | La presencia del episodio depresivo mayor actual será medida por el PHQ-9. [(18)](https://www.zotero.org/google-docs/?4NuMoL)  Puntaje de:   - 0-9 → Episodio depresivo mayor ausente - 10-25 → Episodio depresivo mayor actual | Independiente | Categórica/ Dicotómica | Dicotómica | 0 = Ausente  1 = EDMA | Escala Patient Health Questionnaire - 9  “ |
| **Episodio depresivo mayor actual (3 categorías)** |  | La presencia del episodio depresivo mayor actual será medida por el PHQ-9. [(18)](https://www.zotero.org/google-docs/?4NuMoL)  Puntaje de:   - 0-9 → Episodio depresivo mayor ausente - 10-14 → Episodio depresivo mayor actual leve - 15-25 → Episodio depresivo mayor actual moderado- severo | Independiente | Categórica/ Politomica | Ordinal | 0 = Ausente  1 = Leve  2 = Moderado- severo | Escala Patient Health Questionnaire - 9 |
| **Amenorrea secundaria** | La ausencia de la menstruación al menos de tres ciclos consecutivos, o por un período de 6 meses en mujeres que previamente han menstruado | La presencia de amenorrea secundaria se evaluará por la pregunta basada en el cuestionario de Shapley. M:   - “En los últimos 6 meses, ¿ha dejado de menstruar? → Sí (presencia de amenorrea secundaria)” | Dependiente | Categórica/ Dicotómica | Nominal | 0 = Sí  1 = No | Cuestionario basado en el estudio realizado por Shapley. M et al en Reino Unido en 2004, el cual fue publicado en la revista *British Journal of General Practice*. Evaluado por juicio de expertos |
| **Oligomenorrea** | Trastorno en el que la duración del ciclo es mayor de lo habitual, de manera que las menstruaciones se suceden con intervalos de 35-90 días. | La presencia de oligomenorrea se evaluará por la pregunta basada en el cuestionario de Shapley. M:   - “En los últimos 6 meses, ¿ha tenido períodos menstruales con más de 35 días entre uno y otro? → Sí (presencia de oligomenorrea)” | Dependiente | Categórica/ Dicotómica | Nominal | 0 = No  1 = Sí |  |
| **Polimenorrea** | Trastorno que consiste en el acortamiento del intervalo menstrual a menos de 21 días. | La presencia de polimenorrea se evaluará por la pregunta basada en el cuestionario de Shapley. M:   - “En los últimos 6 meses, ¿ha tenido períodos menstruales con menos de 21 días entre uno y otro?→ Sí (presencia de polimenorrea)” | Dependiente | Categórica/ Dicotómica | Nominal | 0 = No  1 = Sí |  |
| **Edad** | Grupo etario de 18-30 años | Se realizará la pregunta dirigida a la persona para determinar el grupo etario al que pertenecen y solo incluir a las que pertenezcan a este grupo de edad:   - “¿Cuál es tu edad?” | Covariable | Numérica / Discreta | Razón | Respuesta escrita de la encuestada (años) | Preguntas elaboradas por los autores y validados por un juicio de expertos |
| **Facultad** | Facultad disponible de la Universidad Peruana de Ciencias Aplicadas de la que es parte la encuestada | Se incluirá en la encuesta una pregunta dirigida a determinar la facultad a la que pertenece la encuestada realiza la mayoría de sus estudios:   - “¿A qué facultad perteneces?” | Covariable | Categórica/ Politómica | Nominal | 0 = Administración en hotelería y turismo  1 = Arquitectura  2 = Artes contemporáneas  3 = Ciencias de la Salud  4 = Ciencias humanas  5 = Comunicaciones  6 = Derecho  7 = Diseño  8 = Economía  9 = Educación  10 = Ingeniería  11 = Negocios  12 = Psicología |  |
| **Trastornos relacionados a la salud mental** | Trastorno bipolar  Trastorno límite de personalidad  Trastorno de ansiedad generalizada | Se incluirá una pregunta con el fin de valorar diagnósticos diferenciales que tengan episodios depresivos similares para poder excluirlos y solo valorar a los pacientes sin estas condiciones que pueden afectar los resultados:  -“¿Algún médico le ha diagnosticado alguno de los siguientes trastornos de salud mental: trastorno bipolar, algún trastorno de la personalidad y/o trastorno de ansiedad generalizada?”   - Si → se analizará como covariable - No → no se considera como covariable | Covariable | Categórica /Politómico | Nominal | 0 = No  1 = Sí |  |
| **Enfermedades endocrinas** | Síndrome de ovario poliquístico  Hiperprolactinemia  Menopausia precoz  Hipotiroidismo | Se incluirá en la encuesta una pregunta dirigida para poder excluir al grupo de personas con enfermedades endocrinas, con el objetivo de que no influyan en los resultados del estudio:  -“¿Algún médico le ha diagnosticado alguno de los siguientes trastornos endocrinos: síndrome de ovario poliquístico, hiperprolactinemia, menopausia precoz e hipotiroidismo?”   - Si → se analizará como covariable - No → no se considera como covariable | Covariable | Categórica /Politómica | Nominal | 0 = No  1 = Sí |  |
| **Trastornos de la conducta alimentaria** | Anorexia Nerviosa  Bulimia Nerviosa  Ortorexia | Se incluirá en la encuesta una pregunta dirigida a poder excluir al grupo de personas con estos antecedentes con el objetivo de que no influyan en los resultados del estudio:  - "¿Ha sido diagnosticada por un médico con algún trastorno de la conducta alimentaria (anorexia nerviosa, bulimia nerviosa y ortorexia)?”   - Si → se analizará como covariable - No → no se considera como covariable | Covariable | Categórica /Politómico | Nominal | 0 = No  1 = Sí |  |
| **Deportes de competitividad** | Maratones  Baloncesto  Voley  Fútbol  Boxeo  Judo  Taekwondo | Se incluirá en la encuesta una pregunta dirigida para excluir al grupo de personas que son deportistas destacados:  - “¿Actualmente, practicas algún deporte competitivo de manera profesional (correr maratones, jugar baloncesto, voley o fútbol, o participar en competencias de boxeo, judo o taekwondo)?”   - Si → se analizará como covariable - No → no se considera como covariable | Covariable | Categórica/ Dicotómica | Nominal | 0 = No  1 = Sí |  |
