# Supplementary material for: Association between current major depressive episode and the occurrence of menstrual irregularities in the last six months in university students in Lima, Peru
Source: Rev Peru Med Exp Salud Publica. 2025 Dec 11;42(4):400–8. doi: 10.17843/rpmesp.2025.424.14992 (PMC12879977; doi:10.17843/rpmesp.2025.424.14992)
Supplement: Translation

# Translation: Asociación entre el episodio depresivo mayor actual y la ocurrencia de irregularidades menstruales en los últimos seis meses en estudiantes universitarias en Lima, Perú

**Authors:** Ximena Pantoja-Coronel, Gianella Castillón-Véliz, Valeria Ninanya-Cruz, Gema Roca Terry, Yvana Morales-Sánchez, Fabián Fiestas

**Affiliations:** 1 Universidad de Ciencias Aplicadas, Lima, Peru.

**Keywords:** Disturbio Menstrual, Amenorrea, Oligomenorrea, Trastorno Depresivo Mayor

## Abstract

**Objetivos.:**

Evaluar si existe la asociación entre el episodio depresivo mayor actual (EDMA) y las irregularidades menstruales (IM), incluyendo amenorrea, oligomenorrea o polimenorrea, en estudiantes universitarias peruanas en el 2024.

**Materiales y métodos.:**

Estudio observacional, transversal y analítico, en estudiantes de una universidad privada de Lima, Perú. Se utilizó el Patient Health Questionnaire 9 (PHQ-9) para identificar un EDMA y una encuesta de preguntas cerradas para identificar casos de IM. La medida de asociación fue la razón de prevalencia (RP), derivada de modelos de regresión de Poisson, crudos y ajustados por potenciales factores de confusión.

**Resultados.:**

Participaron 250 mujeres entre 18 y 30 años (mediana de edad: 20 años, rango intercuartil de 19 a 21 años). El 73**,**2% presentó al menos una IM (amenorrea, oligomenorrea o polimenorrea) y el 70**,**8% tuvo EDMA de cualquier grado (29**,**2% leve y 41**,**6% moderado-severo). En comparación con las estudiantes sin EDMA, la ocurrencia de cualquier IM fue más probable en aquellas estudiantes con EDMA moderado-severo, RPa=1**,**33; IC 95%: 1**,**08-1**,**62; p=0**,**006), pero no fue diferente con respecto a aquellas con EDMA leve (PRa=1**,**20; IC 95%: 0**,**95-1**,**5; p=0**,**112).

**Conclusiones.:**

Las IM y el EDMA son frecuentes en estudiantes universitarias. El EDMA moderado-severo estuvo asociado a un mayor riesgo de IM. Futuros estudios longitudinales permitirán establecer la temporalidad y otros aspectos relevantes para valorar una posible relación causal entre las IM y el EDMA, incluyendo la relación específica con cada tipo de IM.

## INTRODUCCIÓN

La irregularidad menstrual (IM) es considerablemente común. A nivel global, la prevalencia de ciclos menstruales irregulares varía entre 5% a 35,6% y varía según la edad, el país de procedencia y la ocupación ^1^. Las irregularidades menstruales más frecuentes son la amenorrea, la oligomenorrea y la polimenorrea ^2^. En el Perú, no se hallaron estudios epidemiológicos acerca de la magnitud de este problema ni de su localización en la experiencia poblacional, en términos de espacio, tiempo y persona.

Por su parte, los trastornos del ánimo y el trastorno depresivo mayor son también problemas de salud prevalentes en el Perú, especialmente entre adolescentes y adultos jóvenes. En 2023, el Ministerio de Salud (MINSA) atendió 280 917 casos de depresión, de los cuales la mayoría fueron mujeres (75,5%) y un 16,5%, menores de 18 años ^3^. Asimismo, un estudio epidemiológico en población general, realizado en el 2005 en cinco ciudades peruanas de la costa, sierra y selva, se encontró que el trastorno depresivo mayor tuvo una prevalencia anual de 2**,**7% entre los adultos, colocando a la depresión entre los trastornos mentales más comunes en el país ^4^. Esta alta prevalencia de la depresión explica el hecho que este trastorno esté entre las primeras 10 causas de carga de enfermedad en el Perú. Específicamente, para el 2019, el Ministerio de Salud peruano ha reportado que la depresión unipolar ha causado entre 150 000 y 200 000 años de vida perdidos por discapacidad o muerte prematura ^5^.

La extensión y relación de la depresión con múltiples problemas de la salud física de la persona, hace de ésta una condición de alta sospecha de contribuir en la vía causal de diversos trastornos físicos crónicos, especialmente aquellos con una alta carga fisiopatológica hormonal subyacente, como son las IM. La relación entre trastorno depresivo mayor e IM cuenta con cierta evidencia empírica. Por un lado, se ha encontrado que durante la depresión se activa el eje hipotalámico-pituitario-adrenal (HPA), lo cual conlleva a la inhibición de las hormonas reproductivas y, como consecuencia, a una alteración en la función ovárica ^6^. A estos hallazgos a nivel hormonal, se añaden los resultados de un estudio realizado en Corea del Sur, donde se encontró que las mujeres con ciclos irregulares tuvieron mayores síntomas depresivos ^7^.

El trastorno depresivo mayor y las irregularidades menstruales han sido estudiadas por separado extensamente, pero la asociación entre ellas ha sido muy escasamente investigada. De hecho, de establecerse una conexión causal entre ambas entidades tendría importantes implicancias tanto en la prevención como en el manejo de estos problemas de salud tan comunes entre las mujeres en edad reproductiva. Así, el presente estudio tuvo el objetivo de evaluar si existe asociación entre la ocurrencia del episodio depresivo mayor actual y las IM en mujeres adultas, jóvenes, estudiantes universitarias peruanas.

**MENSAJE CLAVE**

Motivación para realizar el estudio. La depresión y las irregularidades menstruales (IM) son comunes y es plausible una relación causal entre ambas condiciones dada su naturaleza neuro-hormonal.

Principales hallazgos. El presente estudio encontró que, con respecto a las mujeres sin episodio depresivo mayor (EDMA), aquellas con un EDMA de grado moderado-severo tienen mayor probabilidad de cursar con alguna IM. Por otra parte, las mujeres con EDMA leve no se diferenció de aquellas sin EDMA en la probabilidad de IM.

Implicancias en salud pública. Los resultados apoyan la hipótesis causal, pero se requieren estudios longitudinales para confirmar si la depresión aumenta el riesgo de IM. De corroborarse, mujeres jóvenes con EDMA deberían recibir atención ginecológica preventiva y orientada al manejo de las IM.

## MATERIALES Y MÉTODOS

### Diseño

Se realizó un estudio observacional, transversal y analítico.

### Población

La población de estudio incluyó a las estudiantes de una universidad privada de Lima, Perú en el periodo de mayo a junio de 2024. Los criterios de inclusión fueron tener entre 18-30 años. Se excluyeron a las mujeres gestantes o en período de lactancia, o con amenorrea primaria, y a aquellas usuarias de fármacos como anticonceptivos hormonales, medicamentos antiinflamatorios esteroideos (AINES) y anticoagulantes de forma crónica y constante con un tiempo mínimo de 6 semanas.

### Definición de variables

La variable de exposición es la ocurrencia de episodio depresivo mayor actual (EDMA), medida con la escala Patient Health Questionnaire (PHQ-9). La variable de desenlace es la ocurrencia de alguna irregularidad menstrual, incluyendo amenorrea, oligomenorrea o polimenorrea. Como variables con un potencial efecto confusor, se incluyeron a la edad, carrera universitaria y antecedente de diagnóstico de algún trastorno de salud mental (trastorno bipolar, trastorno límite de personalidad y trastorno de ansiedad generalizada). Otras variables confusoras consideradas fueron la presencia de algún trastorno endocrino (síndrome de ovario poliquístico, hiperprolactinemia, menopausia precoz e hipotiroidismo), de algún trastorno de la conducta alimentaria (anorexia nerviosa, bulimia nerviosa y ortorexia); y si la mujer practica algún deporte competitivo (material suplementario).

Para la variable independiente, o de exposición, se realizó una suma del puntaje obtenido por cada ítem del PHQ-9. A partir de ello, se generaron dos tipos de variables, una dicotómica y la otra de tres categorías. La variable dicotómica identificó a aquellas que no presentaban el EDMA (codificado como cero) y a aquellas que sí (codificada como 1). Por su parte, la variable donde la EDMA se clasifica en tres categorías incluyó la categoría sin EDMA (codificada como cero), la categoría de EDMA leve (codificada como 1) o y la de EDMA moderado-severo (codificada como 2). Para la variable dependiente o de desenlace, si una participante respondía «Sí» a cualquiera de las preguntas que codificaban amenorrea, oligomenorrea y polimenorrea, se consideraba como IM presente, codificándose como 1, y 0 si no reportaba ninguna de las tres condiciones.

Para una mejor comprensión de la definición y categorización de cada variable, se puede consultar el material suplementario.

### Muestra

El cálculo muestral se ha realizado con el programa EPIDAT 4.2. La variable dependiente se tomó como una variable categórica dicotómica de escala nominal, por lo que se realizó una comparación de proporciones independiente. Para realizar el cálculo tomamos datos del estudio de Maurya *et al*. (2022), el cual encontró que de 1334 mujeres que tenían irregularidades menstruales, 376 (28%) tenían depresión. Además, se encontró que de 11 373 mujeres que no tenían irregularidades menstruales, 1638 (14%) tenían depresión ^8^. Para el cálculo apropiado, utilizamos un nivel de confianza del 95% y una potencia del 80%. Aunque el cálculo del tamaño de la muestra resultó con estos supuestos en 264 personas con 132 para cada grupo, se logró reclutar finalmente 250 personas con encuestas válidas. El tipo de muestreo fue no probabilístico por conveniencia.

### Instrumentos

El cuestionario, presentó 19 preguntas que fueron divididas en dos secciones: sección 1: datos generales (edad y facultad), irregularidad menstrual (amenorrea, oligomenorrea o polimenorrea) y factores de confusión (antecedente de diagnóstico de algún trastorno de salud mental, de algún trastorno endocrino, de algún trastorno de la conducta alimentaria y la práctica de algún deporte competitivo en la actualidad); y sección 2: episodio depresivo mayor actual.

Para la sección de las IM, se usaron los ítems del cuestionario realizado por Shapley *et al*. (2004) para identificar la ocurrencia de oligomenorrea, polimenorrea y amenorrea. Las preguntas usadas fueron las siguientes: «En los últimos 6 meses, ¿ha tenido períodos menstruales con menos de 21 días (3 semanas) entre un período y otro?», para polimenorrea; «En los últimos 6 meses, ¿ha tenido períodos menstruales con más de 35 días (5 semanas) entre un periodo y otro?», para oligomenorrea; y «¿Ha tenido un período menstrual en los últimos 6 meses?», para amenorrea ^9^. Esta sección del cuestionario fue sometida, para los propósitos de esta investigación, al juicio de expertos con dos ginecólogos y una endocrinóloga para validar la exactitud de la traducción al castellano. Se les presentó tanto la versión en su idioma original (inglés) como la traducción realizada por el grupo de investigación, para que hicieran la debida comparación. Tras una revisión exhaustiva, en el que verificaron que el significado de la pregunta no se viera alterada al ser traducida al castellano, validaron la traducción del instrumento de forma unánime.

Para la segunda sección, se utilizó la escala PHQ-9, un cuestionario de autoreporte basado en 9 criterios para el trastorno depresivo mayor según la DSM-4, los cuales permanecen vigentes en el DSM-5. Este instrumento evalúa los síntomas depresivos durante las últimas dos semanas, calificándolos en una escala tipo Likert de 4 puntos ^10^^,^^11^. Como se muestra en el anexo 2, para fines del presente estudio, se ha considerado un puntaje de 0 a 9 como «EDMA ausente», de 10 a 14 como «EDMA leve» y de 15 a 27 como «EDMA moderado severo»; considerando que el punto de corte para diagnosticar EDMA es mayor o igual a 10, cuya sensibilidad y especificidad es de 88% ^10^. La fiabilidad de este cuestionario fue validada en Perú por Villarreal-Zegarra *et al.* en 2023, en el que se determinaron buenas propiedades psicométricas en cuánto a validez estructural (índice de ajuste comparativo [CFI]=0**,**974) y consistencia interna (α=0**,**89; ω=0**,**86) ^12^.

### Recolección de datos

Las participantes fueron seleccionadas mediante un muestreo no probabilístico en el campus de una universidad privada en Lima, Perú durante el periodo comprendido entre el 27 de mayo al 6 de junio de 2024. El trabajo de campo fue realizado por cinco investigadoras (XPC, GCV, VNC, GRT y YM), quienes se organizaron en grupos para ingresar a salones de clase, pasadizos y salas de estudio, donde invitaron a los estudiantes a participar en el estudio. A aquellas que mostraron interés se les proporcionó un código QR que dirigía al consentimiento informado. Tras su lectura, los participantes podían acceder al cuestionario únicamente si presionaban el botón «aceptar», indicando así su consentimiento. El equipo de investigación permaneció presente durante el llenado del cuestionario en línea, aunque la participante respondió desde su teléfono móvil propio, confidencialmente. De esta forma, se garantizó la correcta comprensión del cuestionario y una aplicación uniforme entre los participantes. El llenado en línea del cuestionario se realizó en Google Forms y duró aproximadamente 8 minutos.

### Plan de análisis

El análisis de los datos recolectados se realizó mediante el paquete estadístico STATA ® versión 17. Las variables categóricas (trastorno depresivo mayor, amenorrea secundaria, oligomenorrea y polimenorrea) se analizaron mediante frecuencias absolutas y proporciones. Por otra parte, la variable numérica (edad) se analizó mediante medidas de tendencia central (mediana) y la medida de dispersión correspondiente (rango intercuartílico). Los análisis bivariados se realizaron mediante Chi cuadrado y prueba de U de Mann Whitney. Igualmente, los análisis de asociación entre la variable independiente y dependiente se realizaron mediante regresión de Poisson cruda y ajustada, tomando en cuenta el potencial efecto de las variables confusoras. La selección de las variables incluidas en el modelo ajustado se efectuó con base en un criterio teórico, sustentado en la literatura científica y en la plausibilidad biológica de su relación con las variables principales del estudio. Se incluyeron aquellas variables que, según la evidencia previa, podrían actuar como factores de confusión entre IM y EDMA. Se realizaron dos tipos de modelamiento de regresión de Poisson: En el modelamiento tipo 1, tanto crudo como ajustado, la variable depresión fue considerada como dicotómica: con y sin EDMA (según lo planificado cuando se hizo el cálculo del tamaño muestral); y en el modelamiento tipo 2, crudo y ajustado, donde la variable depresión fue considerada con tres categorías: EDMA ausente, EDMA leve y EDMA moderado-severo. Se consideró como significativo un valor de p<0,05. Se realizó una exploración estadística para identificar colinealidad entre las variables consideradas en el modelo ajustado a través del factor de inflación de la varianza (VIF).

### Ética y consentimiento informado

El proyecto de investigación fue aprobado por el comité de ética de la Universidad Peruana de Ciencias Aplicadas (PI 134-24). Se siguieron los principios de no maleficencia, beneficencia, justicia y autonomía. Se les informó a las participantes de forma verbal y escrita que tenían total libertad para retirarse durante el proceso de recolección de datos, sin ningún tipo de consecuencia. Asimismo, las encuestas fueron realizadas de manera anónima.

## RESULTADOS

### Selección de muestra

Se invitó a participar a 266 estudiantes mujeres, de las cuales una rechazó la invitación. Del resto, se excluyó a 14 por ser menores de 18 años y a una por haber completado incorrectamente la encuesta. Así, la muestra quedó conformada por 250 mujeres (figura 1).

**Figura 1:** Flujograma de selección de participantes.

### Características epidemiológicas y clínicas de la muestra

Las características de la muestra se presentan en la tabla 1. La mediana de edad de las participantes fue de 20 años (RIC: 19-21), siendo que el 99% de las mismas tenía entre 18 y 26 años. Con respecto a la facultad de estudios, la mayoría (36%) pertenecieron a las carreras de letras y humanidades (Comunicación, Derecho, Educación, Ciencias Humanas, Diseño y Artes Contemporáneas) seguido de un 30,8% que fueron estudiantes de carreras de la salud (Medicina Humana, Nutrición y Dietética y Terapia Física). En cuanto a los antecedentes, 67 participantes (26**,**8%) reportaron haber tenido en algún momento de su vida el diagnóstico de un trastorno de salud mental (trastorno bipolar, trastorno límite de personalidad y trastorno de ansiedad generalizada) y 69 (27**,**6%) haber tenido el diagnóstico de un trastorno endocrino (síndrome de ovario poliquístico, hiperprolactinemia, menopausia precoz e hipotiroidismo). Además, 32 participantes (12**,**8%) han tenido el diagnóstico de un trastorno de conducta alimentaria y 42 participantes (16**,**8%) practicaban un deporte competitivo al momento de la encuesta. La mayoría de las participantes presentaron al menos una IM en los últimos seis meses, siendo en total 183 (73**,**2%). Dentro de las irregularidades menstruales, la más frecuente fue la polimenorrea, que se presentó en 115 participantes (46%), en segundo lugar, se presentó la oligomenorrea en 108 participantes (43**,**2%) y en tercer lugar la amenorrea en 9 participantes (3**,**6%). En cuanto a la presencia de episodio depresivo mayor actual, evaluado según el PHQ-9, un total de 73 participantes (29**,**2%) no mostraron puntajes que indicaran la presencia de dicho trastorno, 73 participantes (29**,**9%) presentaron puntajes correspondientes a una depresión leve mientras que 104 participantes (41**,**6%) tuvieron un puntaje de moderado severo.

**Tabla 1:** Características de las participantes.

Características		n=250	%
Edad (años) ^a^		20	19,2
Facultad			
	Salud	77	30,8
	Letras y Humanidades	90	36,0
	Ingeniería y Arquitectura	49	19,6
	Economía, Negocios y Administración	34	13,6
Antecedente de diagnóstico de un trastorno de salud mental alguna vez en la vida ^b^		67	26,8
Antecedente de diagnóstico de un trastorno endocrino alguna vez en la vida ^c^		69	27,6
Antecedente de diagnóstico de un trastorno de conducta alimentaria alguna vez en la vida ^d^		32	12,8
Práctica de deporte competitivo actualmente		42	16,8
Cualquier irregularidad menstrual (amenorrea, oligomenorrea o polimenorrea)			
	Sí	183	73,2
	No	67	26,8
Irregularidades menstruales ^e^			
	Amenorrea	9	3,6
	Oligomenorrea	108	43,2
	Polimenorrea	115	46,0
	Polimenorrea y oligomenorrea	48	19,2
	Polimenorrea y amenorrea	1	0,4
Episodio depresivo mayor actual			
	Ausente (PHQ-9 <10)	73	29,2
	Sí, Leve (PHQ-9 de 10-14)	73	29,2
	Sí, Moderado severo (PHQ-9 de 15-25)	104	41,6

a Mediana y rango intercuartílico. La edad mínima fue 18 y la máxima de 29 años.

b Trastorno bipolar, trastorno límite de personalidad y trastorno de ansiedad generalizada.

c Síndrome de ovario poliquístico, hiperprolactinemia, menopausia precoz e hipotiroidismo.

d Anorexia nerviosa, bulimia nerviosa y ortorexia.

e Es posible que se presenten casos con más de una IM en los últimos seis meses, por lo que los porcentajes no sumarán 100%

### Asociación entre IM y EDMA

La tabla 2 muestra una asociación estadísticamente significativa entre el EDMA y cualquier IM (p=0,012). Específicamente, de las participantes que tuvieron EDMA leve, el 72**,**6% tenía al menos uno de los tipos de IM, mientras que esta proporción fue de 81**,**7% en las mujeres que presentaban EDMA moderado-severo. Este porcentaje fue de 61,6% entre las mujeres sin EDMA. Por otra parte, no se encontró asociación entre las IM y el resto de variables independientes como la edad (p=0,582), la carrera universitaria (p=0,160), antecedentes de trastorno de salud mental (p=0,110), antecedente de diagnóstico de trastorno endocrino (p=0,151), antecedente de diagnóstico de trastorno de la conducta alimentaria (p=0,501) y la práctica de deportes competitivos (p=0,631).

**Tabla 2:** Asociación entre irregularidades menstruales en los últimos seis meses y trastorno de depresión mayor actual y covariables.

Variables		Irregularidad menstrual		Valor de p^b^
		No n=67 (%)	Sí n=183 (%)	
Episodio depresivo mayor actual				
	Ausente	28 (38,4)	45 (61,6)	0,012
	Sí, Leve	20 (27,4)	53 (72,6)	
	Sí, Moderado severo	19 (18,3)	85 (81,7)	
Edad (años)		20 (18-21)*	20 (19-21)^a^	0,582
Facultad				
	Salud	27 (35,1)	50 (64,9)	0,160
	Letras y Humanidades	20 (22,2)	70 (77,8)	
	Ingeniería y Arquitectura	14 (28,6)	35 (71,4)	
	Economía, Negocios y Administración	6 (17,7)	28 (83,4)	
Antecedente de diagnóstico de un trastorno de salud mental alguna vez en la vida				
	No	54 (29,5)	129 (70,5)	0,110
	Sí	13 (19,4)	54 (80,6)	
Antecedente de diagnóstico de un trastorno endocrino alguna vez en la vida				
	No	53 (29,3)	128 (70,7)	0,151
	Sí	14 (20,3)	55 (79,7)	
Antecedente de diagnóstico de un trastorno de conducta alimentaria alguna vez en la vida				
	No	60 (27,5)	158 (72,5)	0,501
	Sí	7 (21,9)	25 (78,1)	
Práctica de deporte competitivo actualmente				
	No	57 (27,4)	151 (72,6)	0,631
	Sí	10 (26,8)	32 (73,2)	

a Mediana y rango intercuartílico. La edad mínima fue 18 y la máxima de 29 años.

b Para las variables categóricas se usó la prueba de Chi cuadrado y para la variable numérica edad, se usó la prueba de suma de rangos de Wilcoxon.

En la tabla 3, se muestra los resultados de los análisis de regresión de Poisson, En el modelamiento tipo 1, se encontró que el EDMA estuvo asociado a tener IM, tanto en modelo crudo (RPc=1,26; IC 95%: 1,04‒1,54; p=0,020) como en el ajustado (RPa=1,25; IC 95%: 1,02‒1,53; p=0,034). En el modelamiento tipo 2, no se encontró una asociación estadísticamente significativa entre el EDMA leve e irregularidades menstruales en la regresión simple ni en el modelo ajustado. Sin embargo, se observó que la prevalencia de irregularidades menstruales en las mujeres con síntomas de EDMA moderado-severo fue 1**,**28 veces la prevalencia de la misma entre las mujeres sin dicho trastorno (RP=1**,**33; IC 95%: 1**,**08‒1**,**62; valor p=0**,**006). Esta asociación mantuvo su significancia estadística aún después del ajuste por edad, sexo, antecedente de trastornos mentales, trastornos endocrinos, trastornos de la conducta alimentaria y práctica de deporte competitivo actualmente (RPa=1**,**28; IC 95%: 1**,**03‒1**,**58; p=0**,**022).

**Tabla 3:** Asociación entre episodio depresivo mayor actual e irregularidades menstruales: Modelos crudos y ajustados.

Variable		Asociación cruda			Asociación ajustada		
		RPc	IC (95%)	Valor de p	RPa^a^	IC (95%)	Valor de p
Episodio depresivo mayor actual (dos categorías)							
	Sin depresión	1			1		
	Con depresión	1,26	1,04‒1,54	0,020	1,25	1,02‒1,53	0,034
Episodio depresivo mayor actual (tres categorías)							
	Ausente	1			1		
	Leve	1,78	0,94‒1,48	0,163	1,20	0,95‒1,5	0,112
	Moderado severo	1,33	1,08‒1,62	0,006	1,28	1,03‒1,58	0,022

RPc: razón de prevalencia crudo, RPa: razón de prevalencia ajustado, IC: intervalo de confianza.

a Ajustado por edad, facultad, antecedente de diagnóstico de trastorno de salud mental (trastorno bipolar, trastorno límite de personalidad y trastorno de ansiedad generalizada), de algún trastorno endocrino (síndrome de ovario poliquístico, hiperprolactinemia, menopausia precoz e hipotiroidismo) y de algún trastorno de la conducta alimentaria (anorexia nerviosa, bulimia nerviosa y ortorexia) y práctica de deporte competitivo actualmente.

Exploratoriamente, se valoró también la relación entre la presencia de EDMA y cada una de las tres irregularidades menstruales estudiadas, tomadas independientemente una de la otra (tabla 4). La asociación entre amenorrea y el episodio depresivo mayor actual no fue estimable debido a que el tamaño de la muestra fue limitado, siendo que las nueve mujeres con amenorrea, todas resultaron con EDMA. Por su parte, la oligomenorrea no se encontró asociada al EDMA, tanto en el análisis crudo como en el ajustado. Adicionalmente, se encontró que la proporción de polimenorrea en las mujeres con EDMA leve fue 1**,**5 veces la proporción de la misma entre las mujeres sin dicho trastorno. Esta asociación se mantuvo estadísticamente significativa aún después del ajuste por los potenciales factores de confusión (RPa=1**,**6; IC 95%: 1**,**0‒2**,**3; p=0**,**032). Por último, se encontró una asociación entre las mujeres con EDMA moderado severo y la presencia de polimenorrea en la regresión simple (RP=1**,**6; IC 95%: 1**,**1‒2**,**3; p=0**,**018). Sin embargo, esta asociación no se mantuvo estadísticamente significativa a un alfa igual a 0**,**05, probablemente por un tamaño de muestra insuficiente para sostener el modelo multivariado (RPa=1**,**5; IC 95%: 0**,**9‒2**,**2; p=0**,**066). Para todos los modelos, el VIF resultó ser menor a 2 para todas las variables consideradas en los modelos multivariados.

**Tabla 4:** Asociación entre episodio depresivo mayor actual y oligomenorrea y polimenorrea: Modelos crudos y ajustados

	Variable	Asociación cruda			Asociación ajustada		
		RPc	IC (95%)	Valor de p	RPa^a^	IC (95%)	Valor de p
Oligomenorrea	Episodio depresivo mayor actual						
Ausente	1	-	-	1	-	-
Leve	1	0**,**7‒1**,**5	1	1	0**,**7‒1**,**5	0**,**874
Moderado severo	1**,**1	0**,**8‒1**,**6	0**,**510	1**,**1	0**,**7‒1**,**5	0**,**761
Polimenorrea	Episodio depresivo mayor actual						
	Ausente	1	-	-	1	-	-
	Leve	1**,**5	1**,**0‒2**,**3	0**,**034	1**,**6	1**,**0‒2**,**3	0**,**032
	Moderado severo	1**,**6	1**,**1‒2**,**3	0**,**018	1**,**5	0**,**9‒2**,**2	0**,**066

RPc: razón de prevalencia crudo, RPa: razón de prevalencia ajustado, IC: intervalo de confianza.

a Ajustado por edad, facultad, antecedente de diagnóstico de trastorno de salud mental (trastorno bipolar, trastorno límite de personalidad y trastorno de ansiedad generalizada), de algún trastorno endocrino (síndrome de ovario poliquístico, hiperprolactinemia, menopausia precoz e hipotiroidismo) y de algún trastorno de la conducta alimentaria (anorexia nerviosa, bulimia nerviosa y ortorexia) y práctica de deporte competitivo actualmente.

## DISCUSIÓN

El principal hallazgo de este estudio es que, en comparación con las mujeres universitarias sin EDMA, la ocurrencia de al menos una IM (amenorrea, polimenorrea u oligomenorrea) en los últimos seis meses fue más frecuente en aquellas con EDMA, independientemente de potenciales variables de confusión. Asimismo, se encontró que la asociación entre el IM y el EDMA se da específicamente para aquellas con EDMA modero-severo y no para aquellas con EDMA leve, siendo que éstas no se diferenciaron en la probabilidad de tener IM de las que no presentaron EDMA.

Adicionalmente, este estudio ha encontrado que la IM más frecuente en la población estudiada fue la polimenorrea. Además, una proporción significativa (70,8%) de las participantes presentaban EDMA, con una mayoría mostrando síntomas moderado-severo. No se encontró asociación entre oligomenorrea ni amenorrea y EDMA de cualquier grado, pero sí entre polimenorrea y el EDMA leve.

Aunque es escasa la cantidad de estudios en este tema, especialmente en Latinoamérica, nuestros resultados son consistentes con investigaciones previas. Por ejemplo, Kim *et al*. (2017) encontraron que el número de problemas menstruales se correlaciona con la depresión ^13^. Dicho estudio se centró en mujeres desertoras de Corea del Norte y refugiadas en Corea del Sur, quienes enfrentaron múltiples estresores como condiciones de salud poco favorables, tortura, detención y arresto durante su huida, además de problemas de adaptación cultural y cambios radicales en su estilo de vida, los cuales pueden haber llevado a niveles elevados de estrés y ansiedad y subsecuentemente a problemas menstruales (como amenorrea, hipomenorrea, menorragia, polimenorragia, oligomenorrea, cambios en la cantidad de flujo menstrual y cambios en la cantidad de coágulos sanguíneos). Entre las limitaciones de dicho estudio está el pequeño tamaño de la muestra (n=126). Similarmente, Jung y Pang (2023), en un estudio desarrollado en la población de Corea del Sur encontró que la suma del puntaje de síntomas depresivos era mayor en mujeres con alguna irregularidad menstrual en comparación a aquellas sin depresión ^7^. Asimismo, Maurya *et al*. (2020) encontraron una asociación entre las IM y la depresión en sus diferentes niveles de gravedad (leve, moderada y severa) ^8^ en una muestra de 12 707 adolescentes de los estados de Uttar Pradesh y Bihar en la India. Así, nuestro estudio, realizado en mujeres adultas jóvenes de un país latinoamericano, con un estilo de vida urbano y homogéneo en lo que respecta al nivel socioeconómico, educativo y cultural, encuentra resultados que apoyan la hipótesis causal entre la depresión y las alteraciones menstruales. Además, nuestros hallazgos dan luces sobre la forma en que puede ocurrir esta relación al encontrar que, al compararse con no tener EDMA, es el EDMA moderado-severo, y no el leve, el que se asocia a tener alguna IM. En este sentido, futuros estudios podrían valorar el rol de aspectos ambientales, como la carga académica, en la relación entre EDMA e IM en estudiantes universitarias.

Sin embargo, a pesar de esta evidencia que relaciona la depresión con problemas del ciclo menstrual, aún es prematuro plantear que una correlación causal realmente existe. De hecho, hay estudios con resultados inconsistentes a lo encontrado en nuestro estudio. Por ejemplo, Bisaga *et al*. (2002), en un estudio realizado en Estados Unidos, no evidenciaron una asociación entre polimenorrea y la presencia de síntomas depresivos ^14^. Además, para interpretar que una asociación estadística entre dos variables es causal, debe establecerse necesariamente la temporalidad con que se relacionan las variables. Esto aún está por explorarse en estudios longitudinales, dado que nuestro estudio tuvo diseño transversal.

Una relación causal entre la depresión y alteraciones del ciclo menstrual es biológicamente plausible. El ciclo menstrual es regulado por el sistema endocrino, y este puede afectarse en los estados depresivos. En la depresión, el estrés persistente activa el eje hipotalámico-pituitario-adrenal, lo que provoca una mayor liberación de CRH, ACTH y cortisol. Este desequilibrio hormonal puede alterar la regulación del ciclo menstrual, afectando su regularidad y función ^6^. Así, el resultado de nuestro estudio es consistente con la hipótesis causal, aunque, dado su diseño transversal, no le fue posible establecer que el EDMA fue previo a la IM, como sería necesario demostrar para sostener que la primera causa la segunda.

En lo que se refiere a la frecuencia de IM, nuestros resultados son consistentes con los estudios realizados por Kim *et al*., donde la irregularidad menstrual con mayor prevalencia fue la polimenorrea ^13^. Esto puede ser explicado ya que el ciclo acortado es más fácilmente reconocido por la población femenina. Otra similitud fue la alta frecuencia del EDMA, el cual concuerda con el estudio de Maldonado *et al*., con una frecuencia del 65,5% en una población similar de estudiantes universitarias ^(^^15^. Esto puede deberse a los factores biológicos y psicosociales, que pueden predisponer a trastornos afectivos en población urbana y universitaria ^16^. Por otra parte, es de notar que nuestros resultados referidos a la frecuencia de IM difieren con lo encontrado en el estudio de Mittiku *et al*. (2022). Estos autores encontraron una prevalencia de irregularidades menstruales (33**,**4%) en las estudiantes de Etiopía ^17^, lo que significa una prevalencia bastante menor a lo encontrado en nuestro estudio (73**,**2%). Esto puede atribuirse a las diferencias socioeconómicas y culturas de ambos países, y a diferencias metodológicas en relación a los criterios para definir IM.

El presente estudio no está exento de limitaciones. Entre las principales se incluye la imposibilidad de establecer la temporalidad entre las variables debido al diseño transversal que impide determinar si la depresión es previa a la IM. Otra limitación se refiere a que, el tamaño de muestra alcanzado fue de 250 personas, lo que es 5% menor a las 264 que estipuló el cálculo muestral original. Siendo que esta brecha es pequeña, no tuvo un impacto grande en el poder estadístico para abordar el objetivo primario de este estudio. Sin embargo, el tamaño muestral sí fue limitado respecto a los análisis estadísticos exploratorios para valorar la asociación entre los niveles de severidad de EDMA y cada una de las tres IM estudiadas**.** Por ejemplo, sólo 9 mujeres reportaron amenorrea, y todas ellas tuvieron EDMA, lo que impidió verificar la asociación estadística entre ambas variables. Asimismo, el diseño transversal con muestreo no probabilístico impone un riesgo de sesgo de selección que limita la validez de los resultados, especialmente en lo referente a la estimación de la prevalencia tanto de IM como de EDMA pues no es una muestra representativa de la población de mujeres universitarias en el Perú. Futuros estudios con diseño longitudinal y mayor tamaño muestral son necesarios para confirmar la temporalidad entre IM y EDMA, controlar por sesgos de selección y además establecer si la relación es específica para uno o más de los tipos de IM. Por otro lado, el uso de encuestas de autorreporte podría introducir sesgos de información y de recuerdo, los cuales podrían reducirse si se recolectan datos en tiempo real por especialistas clínicos de manera sistemática en futuros estudios longitudinales. No obstante, estas limitaciones, nuestro estudio explora un tema escasamente estudiado, especialmente en población femenina latinoamericana, y dado sus hallazgos hace razonable continuar profundizando el estudio de la potencial relación entre depresión y las IM, especialmente con metodologías longitudinales.

En conclusión, las mujeres adultas jóvenes con episodio depresivo mayor actual de grado moderado-severo tienen mayor probabilidad de cursar con alguna irregularidad menstrual en los últimos seis meses en comparación a aquellas sin dicho episodio depresivo. Esto no fue el caso con respecto a las mujeres con depresión leve, quienes tuvieron similar probabilidad de tener alguna IM que las mujeres sin episodio depresivo. Investigaciones futuras con mayor tamaño muestral y diseño longitudinal, permitirán esclarecer aspectos aún pendientes sobre la posible relación causal entre el EDMA y las distintas IM.
